# Identification of the major photodegradant in metronidazole by LC-PDA-MS and its reveal in compendial methods

**DOI:** 10.1038/s41598-022-15625-5

**Published:** 2022-07-08

**Authors:** Mei-Ling Chen, Hong-Xia Xu, Wei-Feng Yuan, Sui-Hong Zhao, Xue Li, Lan-Xin Zhu, Zong-Yong Shen, Yu-Jing Liu, Ming-Juan Wang, Ang Ma, Jos Hoogmartens, Erwin Adams

**Affiliations:** 1 Beijing Sun-Novo Pharmaceutical Research Company Ltd, Yunguyuan, No.79 Shuangying West Road, Changping District, Beijing, 102200 People’s Republic of China; 2 Department of Pharmaceutical and Pharmacological Sciences, Pharmaceutical Analysis, KU Leuven, O&N2, PB 923, Herestraat 49, 3000 Leuven, Belgium

**Keywords:** Mass spectrometry, Medical research, Risk factors, Chemistry

## Abstract

Metronidazole in aqueous solution is sensitive to light and UV irradiation, leading to the formation of *N*-(2-hydroxyethyl)-5-methyl-l,2,4-oxadiazole-3-carboxamide. This is revealed here by liquid chromatography with tandem photo diode array detection and mass spectrometry (LC-PDA-MS) and further verified by comparison with the corresponding reference substance and proton nuclear magnetic resonance (^1^H-NMR). However, in current compendial tests for related substances/organic impurities of metronidazole, the above photolytic degradant could not be detected. Thus, when photodegradation of metronidazole occurs, it could not be demonstrated. In our study, an improved LC method was developed and validated, which includes a detection at a wavelength of 230 nm and optimization of mobile phase composition thereby a better separation was obtained.

## Introduction

**Figure 1:**
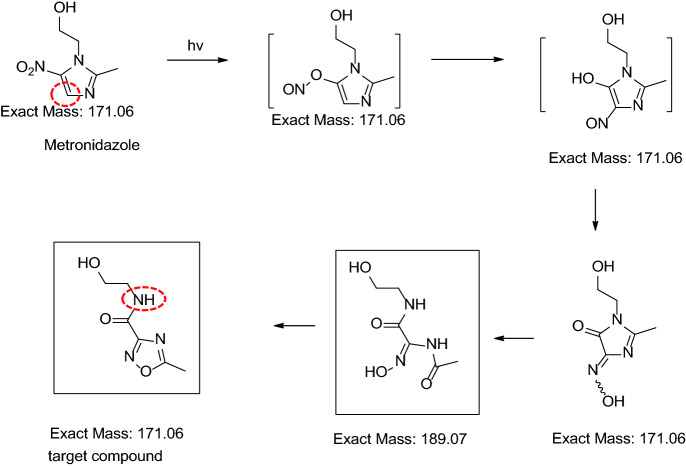
Proposed photolytic rearrangement pathway of metronidazole to *N*-(2-hydroxyethyl)-5-methyl-l,2,4-oxadiazole-3-carboxamide^1–3^.

Metronidazole or 2-(2-methyl-5-nitro-1*H*-imidazol-1-yl)ethanol is a clinically commonly used antibacterial drug with activity against anaerobic infections. However, it is sensitive to light^1–11^ and UV irradiation could lead to its rearrangement and the formation of *N*-(2-hydroxy ethyl)-5-methyl-l,2,4-oxadiazole-3-carboxamide (photolytic degradant, target compound in Fig. 1) via labile intermediates, especially in solution^1–6^.

Metronidazole is a compendial drug substance. In its monographs of European Pharmacopoeia 10 (EP10)^7^, British Pharmacopoeia 2017 (BP2017)^8^, United States Pharmacopeia 43 (USP43)^9^, Japanese Pharmacopoeia XVII (JP17)^10^ and Chinese Pharmacopoeia 2020 (ChP2020)^11^, photosensitization potentials have been emphasized in all the storage and solution preparations.

Unfortunately, in current compendial tests for related substances/organic impurities of metronidazole, the above photolytic degradant can not be detected at the prescribed wavelength (315/319 nm)^7–11^. Thus, when photodegradation of metronidazole occurs, it could not be demonstrated. Up till now, no paper has been published in literature describing a control method for the photodegradation product of metronidazole.

Analysis of light stressed metronidazole solutions revealed a mass imbalance leading to the start of this investigation. Indeed, degradation percentages of metronidazole were much higher than those explained by the degradants detected by current compendial methods.

For the structural characterization of the photolytic degradant of metronidazole, liquid chromatography with tandem mass spectrometry (LC–MS) was applied at first stage due to its high selectivity and sensitivity in the qualification of unknown compounds^12–18^. Further structural confirmation was performed by comparison with the corresponding reference substance (RS) in terms of retention time and extracted photo diode array (PDA) spectra of the peaks obtained in the chromatograms. Finally, proton nuclear magnetic resonance (^1^H-NMR) of the photolytic degradant in CDCl_3_ and D_2_O was performed because of its unambiguous chemical characterization.

## Experimental

### Reagents, standards and samples

Methanol, acetonitrile (MREDA, Beijing, P.R.China) and trifluoroacetic acid (KERMEL, Tianjin, P.R.China) used in this study were of HPLC grade. Water (specific resistance > 18.2 MΩ, total organic carbon (TOC): 0.39 < 0.50 mg/L^19,20^) was obtained from a HHitech water purification system (Shanghai, P.R.China), and other chemicals used were of analytical reagent grade.

Metronidazole RS (100191-201808) and 2-methyl-5-nitroimidazole (impurity I of ChP2020, specified impurity of JP17, tinidazole related compound A of USP43) RS (100512-202005) were obtained from the National Institutes for Food and Drug Control (NIFDC), Beijing, P.R. China. The photolytic degradant of metronidazole, *N*-(2-hydroxyethyl)-5-methyl-l,2,4- oxadiazole-3-carboxamide (CAS:110578-73-9) RS was purchased from Quality Control Chemicals Inc. (Newark, DE, USA).

Light stressed samples of metronidazole drug substance in water (0.2 mg/mL) and its vaginal lotion (containing 10 mg metronidazole and 60 mg chlorhexidine gluconate in 50 mL, with as excipients: polysorbate 80 (1 mg/mL), ethanol (1.0%, v/v) and water) were obtained under UV irradiation of 5000 lx in one Labonce^®^ 500 TPS stability chamber (Beijing, P.R.China) at 25 °C for 30 days.

One sample of metronidazole injection (0.1 g/20 mL) from Wuhan Fuxing Biopharmaceutical Co. (Wuhan, Hubei, P.R. China), and samples of metronidazole with sodium chloride for injection from Shijiazhuang No.4 Pharmaceutical Co. (Shijiazhuang, Hebei, P.R. China) (0.5 g of metronidazole and 0.8 g of sodium chloride per 100 mL) and Shandong Qidu Pharmaceutical Co. (Jinan, Shandong, P.R. China) (0.5 g of metronidazole and 0.9 g of sodium chloride per 100 mL) were purchased from the Chinese market (Beijing, P.R. China). The above metronidazole samples for injection were treated under UV irradiation of 5000 lx in one Labonce^®^ 500 TPS stability chamber (Beijing, P.R.China) at 25 °C for 2 and 5 days, as well as at room light (close to the window) for 48 h, to demonstrate the photodegradation risk of metronidazole injection during administration over several hours^21^.

### LC-PDA experiments

In the stress study of metronidazole in aqueous solutions and the vaginal lotion, as well as in further verification testing of its major photolytic degradant, the LC system (Shimadzu, Darul Khusus, Malaysia) consisted of a binary pump (LC-2030C plus), an autosampler (LC-2030C plus), a photo diode array (PDA) detector (LC-2030C plus) and a LC-2030C plus column oven. Data acquisition, analysis and reporting were performed using Shimadzu LC-Solution software. The starting chromatographic conditions chosen were based on available compendial monographs of metronidazole^7–11^. The Kromasil 100-5 C_18_ column (250 mm × 4.6 mm i.d., 5 μm) (AkzoNobel, Bohus, Sweden) was maintained at 30 °C. Mobile phase A (0.05 mol/L KH_2_PO_4_ in water) and mobile phase B (methanol) were pumped at a total flow rate of 1.0 mL/min. The gradient program (time (min), % B) was set as: (0, 20), (12, 20), (30, 40), (40, 40), (45, 70), (50, 70), (51, 20), (60, 20). Sample solutions were 0.2 mg/mL of metronidazole in methanol–water (20:80, v/v) and the system suitability solution contained 1 μg/mL of metronidazole RS, 2-methyl-5-nitroimidazole RS and *N*-(2-hydroxyethyl)-5-methyl -l,2,4-oxadiazole-3-carboxamide RS, respectively. The injection volume was 10 μL and detection wavelengths were set at 315 nm and 230 nm simultaneously.

### LC-PDA-MS experiments

The chromatographic conditions for identification of the major photolytic degradant (target compound in Fig. 1) of metronidazole in the vaginal lotion were based on the chlorhexidine gluconate/digluconate solution monographs of EP10^22^ and USP43^23^. The LC-PDA-MS system was a Waters Acquity UPLC^®^ (Singapore, Singapore) comprising a binary pump (H-class), auto-sampler (FIN), diode array detector (TUV) and mass spectrometer (Acquity QDa). Data acquisition, analysis and reporting were performed with Empower software (Waters). A Luna C_18_ column (250 mm × 4.6 mm i.d., 5 μm) (Phenomenex, Torrance, CA, USA) was selected according to the Knowledge Database of EDQM (https://extranet.edqm.eu/4DLink1/4DCGI/Web_View/mono/658) and maintained at 30 °C. Solution A contained 0.1% (v/v) trifluoroacetic acid in acetonitrile-methanol (90:10, v/v) and solution B contained 0.1% (v/v) trifluoroacetic acid in water. Ratios of solution A to B (v/v) in mobile phases A and B were (5:95) and (90:10), respectively. The total flow rate was 1.0 mL/min with gradient elution (time (min), % B) as follows (0, 0), (8, 0), (20, 20), (25, 20), (35, 45), (45, 45), (47, 0), (55, 0). Compared to EP10/USP43^22,23^, chromatographic conditions were somewhat adapted in order to improve the selectivity in impurity control of metronidazole and chlorhexidine gluconate in the vaginal lotion.

The mass spectrometric conditions were as follows: electrospray ionization mode positive, probe temperature 600 °C, capillary and cone voltages set at 0.8 kV and 15 V, respectively, flow rate of nitrogen 20 L/min and full scan from 50 to 1200 Da. The acquisition of mass spectra was performed on the light stressed vaginal lotion (5000 lx, 30 days) by injecting 100 μL.

### ^1^H-NMR

Unambigious structural confirmation of the photolytic degradant (CAS:110578-73-9) RS (Quality Control Chemicals Inc., Newark, DE, USA) by ^1^H-NMR in CDCl_3_ and D_2_O were performed on BRUKER PLUS 400 (Ettlingen, Germany) at 400 MHz, in comparison with those of metronidazole RS (National Institutes for Food and Drug Control (NIFDC), Beijing, P.R. China).

## Results

### Method development and validation

In the metronidazole monograph of EP10^7^ and BP2017^8^, a mobile phase is prescribed consisting of 1.36 g/L KH_2_PO_4_–methanol (70:30 v/v), while in USP43^9^ and ChP2020^11^ water–methanol (80:20 v/v) is prescribed. It was found in this study that a composition of 0.05 mol/L KH_2_PO_4_–methanol (80:20 v/v), as initial mobile phase showed a more ideal peak symmetry and better resolution between the peaks due to the photolytic degradant and its neighbouring impurities. Then, gradient elution was introduced for elution of chlorhexidine gluconate and its related substances. The proposed method (see “LC-PDA experiments” section for details) has been validated at a wavelength of 230 nm for its intended use in terms of specificity, sensitivity, accuracy, linearity, precision and robustness (see supplementary materials for details). The photolytic degradant was stable in the proposed methanol–water (20:80, v/v) solvent for at least 33 h at room temperature. No interferences from samples subjected to heat, acid, base or oxidation, known specified impurity (2-methtyl-5-nitro imidazole) or sample matrix were observed. The sensitivity (quantitation limit: 0.05%) met the intended identification threshold of 0.2%^24^, as the maximum daily dose of the vaginal lotion expressed in metronidazole is 20 mg.

### Structural characterization of the photolytic degradant

**Figure 2:**
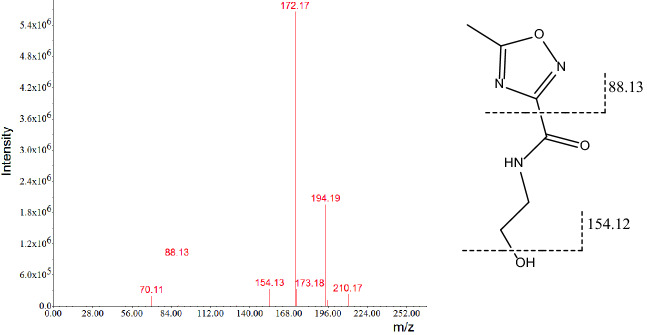
MS spectrum of the major photodegradant of metronidazole. ([M + H]^+^ *m*/*z* 172.17, [M + Na]^+^ *m*/*z* 194.19, [M + K]^+^ *m*/*z* 210.18; and characteristic product ions at *m*/*z* 154.12 and *m/z* 88.13).

**Figure 3:**
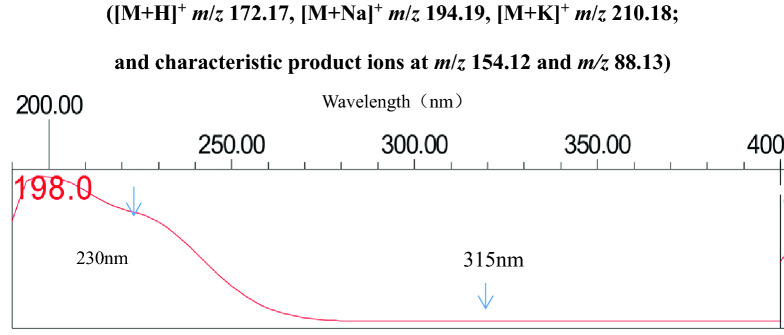
UV spectrum of photolytic degradant of metronidazole.

Photodegradation of metronidazole in the vaginal lotion (about 14% degradation under 5000 lx for 30 days) was detected under the conditions mentioned above. A similar photosensitization trend was observed in aqueous solutions of metronidazole drug substance with ~ 12% loss. Based on the LC-PDA-MS results of the major photolytic degradant of metronidazole ([M + H]^+^ *m*/*z* 172.17, [M + Na]^+^ *m*/*z* 194.19, [M + K]^+^ *m*/*z* 210.18 and characteristic product ions at *m*/*z* 154.12 and *m/z* 88.13 (see Fig. 2), its corresponding UV spectrum (see Fig. 3, with poor absorption at 315 nm) and related references^1–6^, it was identified as *N*-(2-hydroxy ethyl)-5-methyl-l,2,4- oxadiazole-3-carboxamide (CAS:110578-73-9) , whose production pathway was proposed by rearrangement of metronidazole as described in Fig. 1^1–3^. Further verification was performed by comparison of its retention time (4.270 vs 4.272 min) and PDA spectrum with those of its reference substance. It is also worth mentioning that in the MS spectrum the [M + H-46]^+^ (loss of –NO_2_) peak, characteristic for metronidazole at *m*/*z* 126.12, was not observed. Final unambigous structural confirmation of the photolytic degradant by ^1^H-NMR in CDCl_3_ and D_2_O showed that in comparison with the parent molecule metronidazole, loss of the singlet at δ7.94, and replacement by a broad signal at δ7.42 in the photolytic degradant, assigned to the amide proton in the side chain of the oxadiazole. Moreover, the signal at δ7.42 in the photolytic degradant disappears while the signal at δ7.94 in metronidazole remains when D_2_O was added (see supplementary materials for details), totally in agreement with the proposed structure of the target compound in Fig. 1.

### Photodegradation risk evaluation of metronidazole injection

Immediately after opening the packaging, the above photolytic degradant amounted 0.01–0.02% in the two batches of metronidazole with sodium chloride for injection, while it could not be detected in the metronidazole injection without sodium chloride (detection limit: ~ 0.01%). However, its content increased to 0.15% at room light condition for 48 h, and to 0.36% and 0.88% respectively after 2 and 5 days under UV irradation (5000 lx), see supplementary materials for details. These were all higher than its identification threshold and the qualification threshold (maximum daily dose of metronidazole injection: 4 g)^21,24^, demonstrating the necessity to control the photolytic degradant in metronidazole injection, which is typically administered by slow intravenous drip infusion. Unfortunately, the instruction leaflet of marketed metronidazole injections only warned to protect from light during storage while their containers were not light-resistant and no light-protection precautions were proposed during administration^21^.

### Suggestions to improve current compendial methods for related substances of metronidazole and its drug products

**Figure 4:**
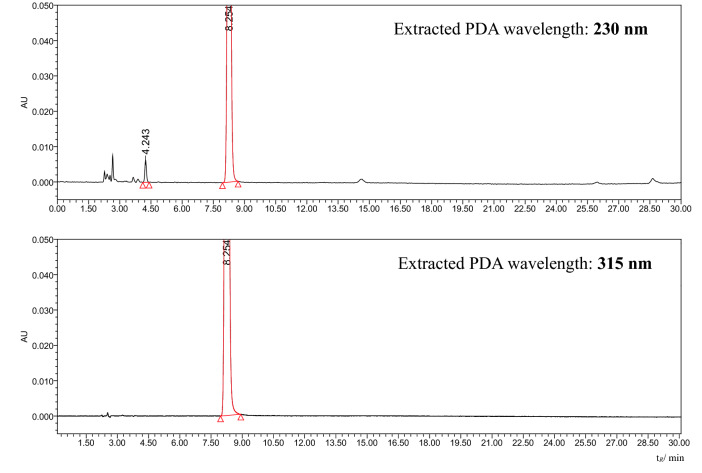
Comparison of chromatograms for light stressed metronidazole in solution (5000 lx, 24 h), with detection at 230 nm and 315 nm, respectively, by LC-PDA experiments.

Based on the above information, it was proposed to modify compendial methods for related substances of metronidazole and its drug products, so the method could be used to monitor the specified impurities and the new photolytic degradant. The proposed method, as described in “LC-PDA experiments” section, has been fully validated for its intended use. The relative correction factor (F) of the photolytic degradant to metronidazole measured at 230 nm is about 0.9, within the acceptable range of 1.0 (0.8–1.2). The major photolytic degradant as well as other minor light stressed degradants could be observed with good sensitivity when the detection wavelength was switched from to 315 to 230 nm, as shown in Fig. 4.

## Discussion

1. Though photosensitization potentials are all emphasized in its storage and solution preparations of metronidazole in EP10, BP2017, USP43, JP17 as well as ChP2020, and real photodegradation was observed in commercial metronidazole injections, especially during its slow intravenous drip infusion, the photolytic degradant could not be detected by current compendial methods for related substances of metronidazole and its products at the described 315/319 nm wavelength. The observation revealed possible reason for the mass imbalance of light stressed metronidazole in solution and its vaginal lotion detected by current compendial methods for its related substances. Thus, suggestions to modify current compendial methods to monitor the new degradant were proposed, e.g. detection at an additional wavelength of 230 nm and modification of the gradient. And other possible precautions against light during its slow intravenous drip infusion of metronidazole injections or similar aqueous products were also suggested. The proposed method has also been fully validated, and its proposed limit (0.2%, maximum daily dose of metronidazole vaginal lotion: 20 mg) was based on ICH Q3B^24^. Maybe further investigations, e.g. its toxicological and pharmacological data, are required to reveal if the proposed limit is scientific and reasonable.
2. The photolytic degradant was found to be stable in the described solvent [methanol–water (20:80, v/v)] for at least 33 h at room temperature in closed, light-protected auto-sampler. This could ensure repeatable determination results. In light stressed metronidazole vaginal lotion (5000 lx, 30 days), besides the photolytic degradant characterized in the study, the intermediate (Mr. 189.07, see Fig. 1) could also be observed, but only in a very small amount (about 8% of the photolytic degradant). It could be observed in the proposed method at a relative retention time (RRT) of ~ 0.44 (the photolytic degradant: RRT =  ~ 0.50). Specified impurity A (2-methtyl-5-nitro imidazole) could also be eluted with RRT ~ 0.68. Other minor light stressed degradants could also be observed, as revealed in Fig. 4 and supplementary materials [Stablity of metronidazole injection under UV irradiation (5000 lx)].
3. In the structural characterization of the photolytic degradant in light stressed metronidazole samples (5000 lx, 30 days), based on reports^1–6^ and our LC-PDA-MS results, it was proposed as *N*-(2-hydroxyethyl)-5-methyl-l,2,4-oxadiazole-3-carboxamide (CAS:110578-73-9). Its typical parent ions, characteristic product ions as well as extracted PDA spectrum were totally in agreement with those of references^1–6^. Further verification was performed by comparison with the corresponding reference substance, in terms of retention times and extracted PDA spectra under the same chromatographic condition (see “LC-PDA experiments” section), which were consistent with each other. The characteristic differences between the ^1^H-NMR data of the photolytic degradant RS and metronidazole RS in CDCl_3_ and D_2_O (see supplementary materials) were consistent with reports^1–6^ and its proposed structure as shown in Fig. 1, which further ensured reliability of the above structural characterization results of the degradant in light stressed metronidazole samples. If necessary, conducting photostress of metronidazole on large scale, then isolation/purification of the degradant maybe are required.

## Supplementary Information

Supplementary Information.

## Data Availability

See the hyperlinks under the Figs. 1, 2, 3, 4 and References.
