# Supplementary material for: Identification of the major photodegradant in metronidazole by LC-PDA-MS and its reveal in compendial methods
Source: Sci Rep. 2022 Jul 8;12:11665. doi: 10.1038/s41598-022-15625-5 (PMC9270378; doi:10.1038/s41598-022-15625-5)
Supplement: Supplementary file 1 — Supplementary Information. [file 41598_2022_15625_MOESM1_ESM.pdf]

Identification of the major photodegradant in metronidazole by LC-PDA-MS and its reveal in compendial methods

Mei-Ling CHEN[a], Hong-Xia XU[a], Wei-Feng YUAN[a], Sui-Hong ZHAO[a], Xue LI[a], Lan-Xin ZHU[a], Zong-Yong SHEN[a], Yu-Jing LIU[a], Ming-Juan WANG[a*], Ang MA[a], Jos HOOGMARTENS[b], Erwin ADAMS[b*]

[a] *Beijing Sun-novo Pharmaceutical Research Company Ltd, Yunguyuan, No.79 Shuangying West Road, Changping District, 102200 Beijing, P.R. China*

[b] *KU Leuven, Department of Pharmaceutical and Pharmacological Sciences, Pharmaceutical Analysis, O&N2, PB 923, Herestraat 49, B-3000 Leuven, Belgium*

Supplementray materials:

(a) Method validation:

（Proposed method for determinnation of the photolytic degradant was described in **LC-PDA experiments** section of the manuscript, at a wavelehgth of **230 nm**）

<table>
<tr><th>Characteristics</th><th>Acceptable criteria</th><th colspan="3">Validation results</th></tr>
<tr><td rowspan="3">SST
(System suitability test)</td><td>Resolution (Rs) : Rs between the photolytic degradant and metronidazole should be greater than 2.0 *</td><td colspan="3">Rs：17.518</td></tr>
<tr><td>Repeatability: Inject diluted sample solution (0.2%) five times, RSD (Relative standard deviation) of retention times (t_R) and peak areas (Area) should be less than 1.0% and 5.0%, respectively</td><td colspan="3">RSD (t_R, %)： 0.6%
RSD (Area, %)：1.0%</td></tr>
<tr><td>Sensitivity: S/N (Signal-to-Noise ratio) of sensitivity solution (0.06%) should be greater than 10</td><td colspan="3">S/N：21.11</td></tr>
<tr><td rowspan="4">Accuracy</td><td rowspan="4">Spiked recovery at 50%, 100% and 150% concentrations of proposed limit should be within 90% ~ 110% (3 replicates at each concentration), and RSD of their spiked recovery should be less than 5%</td><td>Spiked concentration</td><td>Spiked Recovery (%)</td><td>RSD%</td></tr>
<tr><td>50%</td><td>99.0% ~ 104.1%</td><td rowspan="3">1.7%
(n = 9)</td></tr>
<tr><td>100%</td><td>98.6% ~ 100.6%</td></tr>
<tr><td>150%</td><td>98.6% ~ 100.8%</td></tr>
<tr><td>Precision</td><td>RSD obtained in</td><td colspan="3">Repeatability (repeatable injections):</td></tr>
</table>

<table>
<tr><th>Characteristics</th><th>Acceptable criteria</th><th>Validation results</th></tr>
<tr><td rowspan="2"></td><td rowspan="2">repeatability and intermediate precision at 100% spiked concentration should be less than 5% and 15%</td><td>Average：0.26% (operator 1)/0.24% (operator 2);
RSD: 2.1% (operator 1) / < 0.5% (operator 2)（n=6）</td></tr>
<tr><td>Intermediate precision: (two operators, conducted at different days with different LC instruments)
Average：0.25%
RSD: 3.7%（n=12）</td></tr>
<tr><td>Specificity</td><td colspan="2">No interferences from samples subjected to heat (105 ℃, 24 h) , acid (1M HCl, 24 h), base (1M NaOH, 24 h) or oxidation (30% H_2O_2, 24 h) , known impurity A (2-methtyl-5-nitro imidazole) (RRT ~ 0.70) or sample matrix were observed to determination of the photolytic degradant (RRT ~ 0.50).</td></tr>
<tr><td>Limit of detection
(LOD)</td><td>Based on S/N at about 3:1</td><td><table><tr><td>Compounds</td><td>LOD（%）</td><td>S/N</td></tr><tr><td>photolytic degradant</td><td>0.015</td><td>7.17</td></tr><tr><td>metronidazole</td><td>0.016</td><td>5.37</td></tr></table></td></tr>
<tr><td rowspan="3">Limit of quantitation
(LOQ)</td><td>Based on S/N at about 10:1</td><td><table><tr><td>Compounds</td><td>LOQ（%）</td><td>S/N</td></tr><tr><td>photolytic degradant</td><td>0.048</td><td>23.06</td></tr><tr><td>metronidazole</td><td>0.054</td><td>14.18</td></tr></table></td></tr>
<tr><td colspan="2">Proposed limit of the photodegradant was 0.2% (ICH Q3B)</td></tr>
<tr><td>Precision at LOQ concentration: RSD of retention times (t_R) and peak areas (Area) should be less than 1.0% and 5.0%, respectively</td><td><table><tr><td>Compounds</td><td>RSD (t_R, %)</td><td>RSD (Area, %)</td></tr><tr><td>photolytic degradant</td><td>0.1%</td><td>3.5%</td></tr><tr><td>metronidazole</td><td>0.1%</td><td>3.1%</td></tr></table></td></tr>
<tr><td>Linearity</td><td>$r \geq 0.999\ 0$</td><td><table><tr><td>Compounds</td><td>linear equations</td><td>Range (μg/ml)</td><td>r</td></tr><tr><td>degradant</td><td>y = 13765 x - 16</td><td>0.10 ~ 0.78</td><td>0.999 9</td></tr><tr><td>metronidazole</td><td>y = 11921 x - 106</td><td>0.11~ 0.85</td><td>0.999 7</td></tr></table></td></tr>
<tr><td rowspan="2">F (correction factor)</td><td>Regards F as 1.0, when it is withn the range of 0.8~1.2</td><td><table><tr><td>Compounds</td><td>F</td></tr><tr><td>Photolytic degradant</td><td>0.9</td></tr><tr><td>metronidazole</td><td>/</td></tr></table></td></tr>
<tr><td colspan="2">The F value of the photolytic degradant is 1.0 (measured at 230 nm)</td></tr>
<tr><td>Stability of solutions</td><td colspan="2">Spiked sample solution (0.2%) and diluted sample solution (0.2%) were both stable at room temperature for at least 33 h, injected in a closed, light-protected auto-sampler.</td></tr>
</table>

		Variations in method parameters	Results
**Robustness**	RSD of results measured at deliberate variations conditions should be less than 15%	Normal condition (flow rate: 1.0 ml/min, column: Kromasil C_{18}, column temperature: 30℃)	0.24%
		0.8 ml/min	No significant changes were observed for the results when column, column temperature and flow rate were deliberately varied in the measured range （results: 0.23%~0.25%, RSD: 2.7%）
		1.2 ml/min	
		25℃	
		35℃	
		Waters XBridge C_{18}, (250 mm × 4.6 mm, 5 μm)	

* Resolution between the photolytic degradant (RRT ~ 0.50) and known impurity A (RRT ~ 0.70) , or that between impurity A (RRT ~ 0.70) and metronidazole (RRT 1.0) maybe would be considered as critical chromatographic parameters of our proposed method in future study.

(b) ^{1}H-NMR data of the photolytic degradant RS and metronidazole RS in $CDCl_3$ and D_2O

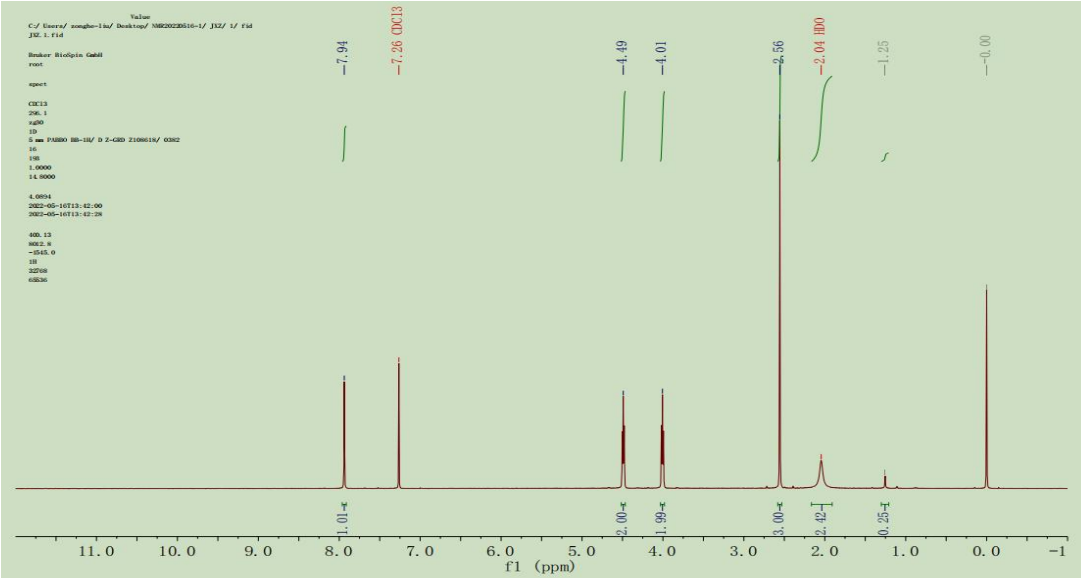

^{1}H-NMR data of metronidazole RS in $CDCl_3$

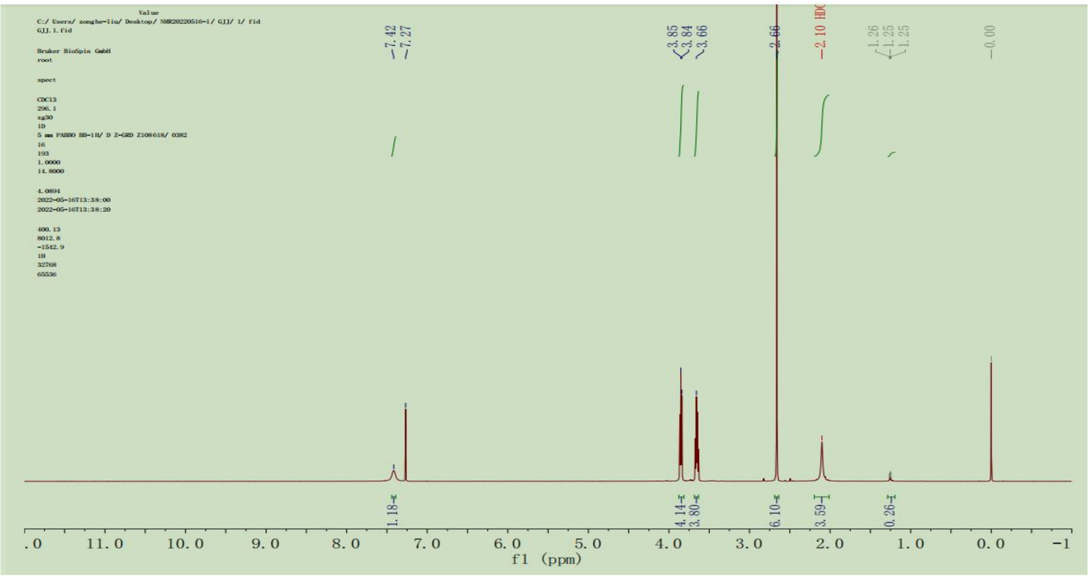

^{1}H-NMR data of the photolytic degradant (CAS:110578-73-9) RS in $CDCl_3$

(Note: The photolytic degradant (CAS:110578-73-9) RS was purchased from Quality Control Chemicals Inc. (Newark, DE, USA) and metronidazole RS (100191-201808) was obtained from the National Institutes for Food and Drug Control (NIFDC), Beijing, PR China.)

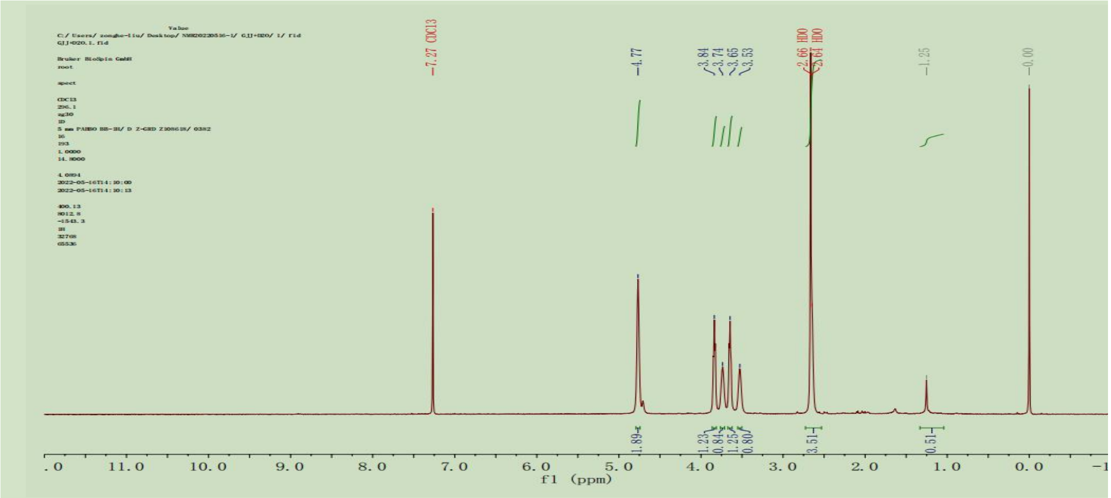

^{1}H-NMR data of the photolytic degradant (CAS:110578-73-9) RS when D_2O was added

(the signal at δ7.42 in the degradant disappears when D_2O was added)

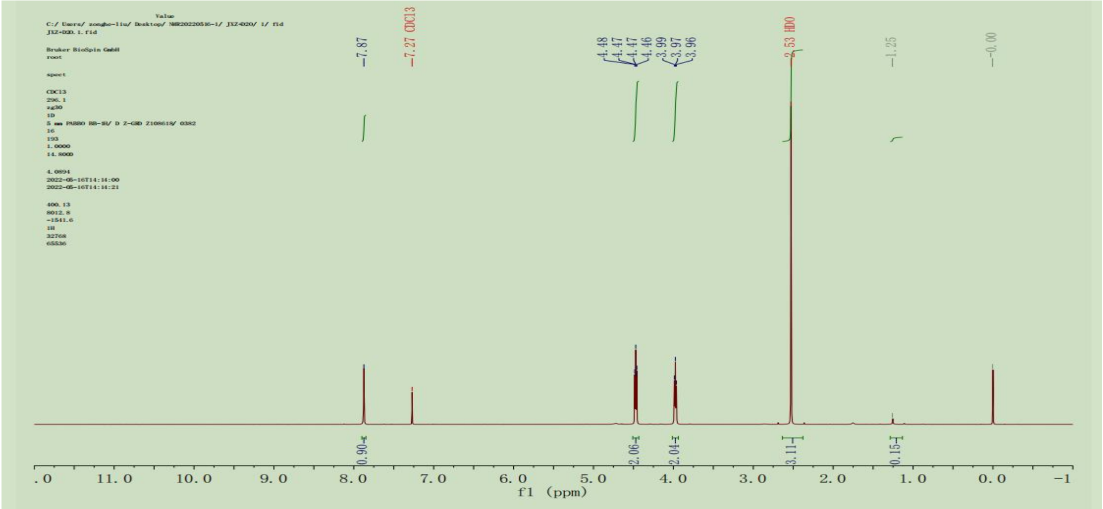

^{1}H-NMR data of metronidazole RS when D_2O was added

(the signal at δ7.94 in metronidazole remains when D_2O was added)

The structural confirmation of the photolytic degradant by ^{1}H-NMR in $CDCl_3$ and D_2O showed that in comparison with the parent metronidazole, loss of the singlet at δ7.94, and replacement by a broad signal at δ7.42 in the photolytic degradant, assigned to the amide proton in the side chain of the oxadiazole. Moreover, the signal at δ7.42 in the photolytic degradant disappears while the signal at δ7.94 in metronidazole remains when D_2O was added, totally in agreement with the proposed structures shown in Fig.1, which ensured reliability of the structural characterization results of the photolytic degradant in light stressed samples by LC-PDA-MS and further verification by comparison with its corresponding RS.

(c) Stability of metronidazole injection under UV irradation (5000 Lux)

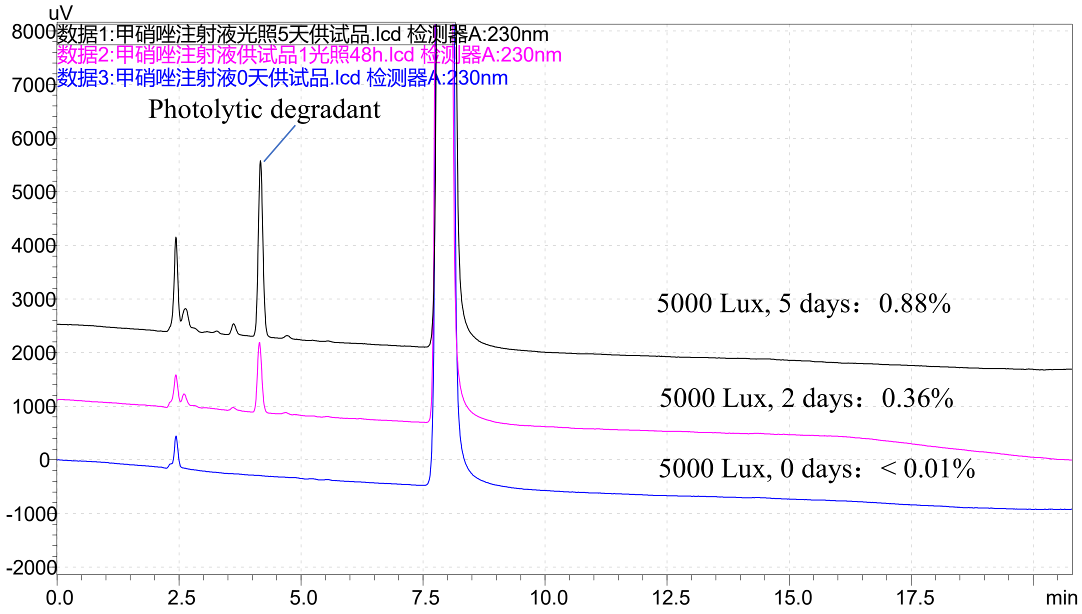

As indicated in the above overlay chromatogram, the photolytic degradant could not be detected in the metronidazole injection without sodium chloride (detection limit: ~0.01%). However, its content increased to 0.36% and 0.88%, respectively after 2 and 5 days under UV irradation (5000 Lux), all higher than its identification threshold (0.10%) and the qualification threshold (0.15%) (maximum daily dose of metronidazole injection: 4 g) [1, 2], demonstrating the necessity to control the photolytic degradant in metronidazole injection, which is typically administered by slow intravenous drip infusion [1].

References

[1] https://www.accessdata.fda.gov/drugsatfda_docs/label/2021/018890s054lbl.pdf (accessed on April 12, 2022)

[2] ICH harmonised tripartite guideline. Q3B: Impurities in new drug products (2006), Geneva, Switzerland.
